# Supplementary material for: Stimulus-induced gamma rhythms are weaker in human elderly with mild cognitive impairment and Alzheimer’s disease
Source: eLife. 2021 Jun 8;10:e61666. doi: 10.7554/eLife.61666 (PMC8238507; doi:10.7554/eLife.61666)
Supplement: Supplementary file 1. — Supplementary table 1: Criteria used for consensus diagnosis of dementia. ACE: Addenbrooke's Cognitive Examination (So et al., 2018); CDR: Clinical Dementia Rating (Hughes et al., 1982; Morris, 1993); GPCOG: General Practitioner Assessment of Cognition (Brodaty et al., 2002); HAMD: Hamilton Depression Rating Scale (Hamilton, 1960; Williams, 1988); HMSE: Hindi Mental State Examination (Ganguli et al., 1995); IADL: Instrumental Activities of Daily Living (Mathuranath et al., 2005); NIA-AA: National Institute on Aging-Alzheimer’s Association workgroups (McKhann et al., 2011); NPI: Neuropsychiatric Inventory (Cummings et al., 1994); TLSA: Tata Longitudinal Study of Aging. 1NIA-AA criteria are presented from McKhann et al., 2011. 2Cognitive decline could also be seen in delirium. So, this criterion is intended to rule out delirium. A combination of clinician assessment and HMSE is used. Acute change in cognitive status and/or HMSE <24 is suggestive of delirium. 3This criterion is intended to rule out moderate/severe depression as that can cause cognitive impairment. Supplementary table 2. Criteria used for consensus diagnosis of probable AD. WMH: white matter hyperintensities; FTD: Frontotemporal dementia. The rest of the abbreviations are as described in Supplementary table 1. Supplementary table 3. Criteria used for consensus diagnosis of MCI. 1Q4. Have there been some decline in memory in the past one year? (Hughes et al., 1982; Morris, 1993). Abbreviations are as described in Supplementary table 1. [file elife-61666-supp1.docx]

**Supplementary file 1: Tables for consensus diagnosis**

**Title: Stimulus-induced gamma rhythms are weaker in human elderly with Mild Cognitive Impairment and Alzheimer’s Disease**

**Running title:** Stimulus-induced **g**amma rhythms weaken in MCI/AD subjects

**Authors:** Dinavahi V. P. S. Murty, Keerthana Manikandan, Wupadrasta Santosh Kumar, Ranjini Garani Ramesh, Simran Purokayastha, Bhargavi Nagendra, Abhishek M. L., Aditi Balakrishnan, Mahendra Javali, Naren Prahalada Rao and Supratim Ray.

**Supplementary Table 1: Criteria used for consensus diagnosis of dementia**

| **NIA-AA criteria for dementia^1^** | **TLSA Criteria for dementia** |
| --- | --- |
| Interfere with the ability to function at work or at usual activities | CDR box scores in the subdomains of outdoor activities, hobbies and personal care: average > 0.5. |
| Represent a decline from previous levels of functioning and performing | GPCOG informant subdomain > 4 |
| Are not explained by delirium or major psychiatric disorder | Clinician’s judgement (no mention of acute change in cognition status during clinical history taking) and/or HMSE > 24^2^ |
|  | HAMD (<14)^3^ |
| Cognitive impairment is detected and diagnosed through a combination of   - History-taking from the patient and a knowledgeable informant - An objective cognitive assessment, either a “bedside” mental status examination or neuropsychological testing | **Any 2 of the following:** |
|  | GPCOG informant subdomain > 4 |
|  | ACE < 88 |
|  | HMSE < 27 |
|  | GPCOG patient subdomain >5 |
|  | Clinician assessment based on sub scores on ACE, HMSE or GPCOG patient |

(Continued…)

| The cognitive or behavioral impairment involves a minimum of two of the following domains   - Impaired ability to acquire and remember new information - Impaired reasoning and handling of complex tasks, poor judgment - Impaired visuospatial abilities - Impaired language functions - Changes in personality, behavior, or comportment | **Any 2 of the following:** |
| --- | --- |
|  | IADL shopping ( >/= 1) |
|  | IADL finance ( >/= 1) |
|  | IADL travel ( >/= 1) |
|  | IADL social ( >/= 1) |
|  | IADL prayer activity ( >/= 1) |
|  | NPI (Any one of the following behavioral problem should be present: apathy, disinhibition, elation, anger) |

ACE: Addenbrooke's Cognitive Examination (So et al., 2018)

CDR: Clinical Dementia Rating (Hughes et al., 1982; Morris, 1993)

GPCOG: General Practitioner Assessment of Cognition (Brodaty et al., 2002)

HAMD: Hamilton Depression Rating Scale (Hamilton, 1960; Williams, 1988)

HMSE: Hindi Mental State Examination (Ganguli et al., 1995)

IADL: Instrumental Activities of Daily Living (Mathuranath et al., 2005)

NIA-AA: National Institute on Aging-Alzheimer’s Association workgroups (McKhann et al., 2011)

NPI: Neuropsychiatric Inventory (Cummings et al., 1994)

TLSA: Tata Longitudinal Study of Aging

^1^NIA-AA criteria are presented from McKhann et al. (2011)

^2^Cognitive decline could also be seen in delirium. So, this criterion is intended to rule out delirium. A combination of clinician assessment and HMSE is used. Acute change in cognitive status and/or HMSE<24 is suggestive of delirium.

^3^This criterion is intended to rule out moderate/severe depression as that can cause cognitive impairment.

**Supplementary Table 2: Criteria used for consensus diagnosis of probable AD**

| **NIA-AA criteria for probable AD** | **TLSA criteria for probable AD** |
| --- | --- |
| Meets criteria for dementia (see Supplementary Table 1) | Meets criteria for dementia (as in Supplementary Table 1) |
| Clear-cut history of worsening of cognition by report or observation | GPCOG informant subdomain > 4 |
| Most prominent cognitive deficits are   - Amnestic presentation - Non-amnestic presentations - Language/visuospatial/executive dysfunction | Clinician’s assessment based on sub scores of ACE or HMSE or GPCOG patient scores |
| Should not have   - Substantial concomitant cerebrovascular disease (Stroke/extensive infarcts/severe WMH) - Features of Lewy body dementia Features of FTD - Features of primary progressive aphasia - Concurrent neurological cause/medical comorbidity/medication impairing cognition | No Clinical history of stroke |
|  | No structural abnormality, no evidence of severe white matter hyperintensities and no evidence of extensive infarcts or bleed, as seen in MRI |
|  | NPI (>2 of the following behavioral problems should be absent: apathy, disinhibition, elation and anger) |
|  | Clinician’s assessment on reversible causes of dementia/other causes of dementia |

WMH: white matter hyperintensities

FTD: Frontotemporal Dementia

The rest of the abbreviations are as described in Supplementary Table 1.

**Supplementary Table 3: Criteria used for consensus diagnosis of MCI**

| **NIA-AA criteria for MCI** | **TLSA criteria for MCI** |
| --- | --- |
| Concern regarding a change in cognition | CDR = 0.5 |
| Impairment in one or more cognitive domains   - Memory - Executive function - Attention - Language - Visuospatial skills | **Any one of the following:** |
|  | GPCOG patient subdomain >5 |
|  | ACE < 88 |
|  | HMSE < 27 |
|  | Clinician’s assessment based on sub scores of ACE/HMSE/GPCOG patient |
| Preservation of independence in functional abilities | IADL (average of finances, shopping, phone and meal preparation <0.5) |
| Not demented | CDR total score (<1) |
| Longitudinal decline in performance over repeated measures | ‘Yes’ for question no. 4^1^ of CDR informant: memory subdomain **or** clinician assessment |

^1^Q4: Have there been some decline in memory in the past one year? (Hughes et al., 1982; Morris, 1993)

Abbreviations are as described in Supplementary Table 1.
