# Supplementary material for: Stimulus-induced gamma rhythms are weaker in human elderly with mild cognitive impairment and Alzheimer’s disease
Source: eLife. 2021 Jun 8;10:e61666. doi: 10.7554/eLife.61666 (PMC8238507; doi:10.7554/eLife.61666)
Supplement: Transparent reporting form [file elife-61666-transrepform.docx]

***eLife’s* transparent reporting form**

We encourage authors to provide detailed information *within their submission* to facilitate the interpretation and replication of experiments. Authors can upload supporting documentation to indicate the use of appropriate reporting guidelines for health-related research (see [EQUATOR Network](http://www.equator-network.org/%20)), life science research (see the [BioSharing Information Resource](https://biosharing.org/)), or the [ARRIVE guidelines](http://www.plosbiology.org/article/info:doi/10.1371/journal.pbio.1000412) for reporting work involving animal research. Where applicable, authors should refer to any relevant reporting standards documents in this form.

If you have any questions, please consult our Journal Policies and/or contact us: [editorial@elifesciences.org](mailto:editorial@elifesciences.org).

**Sample-size estimation**

- You should state whether an appropriate sample size was computed when the study was being designed
- You should state the statistical method of sample size computation and any required assumptions
- If no explicit power analysis was used, you should describe how you decided what sample (replicate) size (number) to use

Please outline where this information can be found within the submission (e.g., sections or figure legends), or explain why this information doesn’t apply to your submission:

Explicit power analysis was not performed prior to the study as this study is part of a large multi-investigator project that involved use of a large number of subjects recruited from the community. We were blind to their diagnosis during recruitment and performed all analyses post-hoc.

**Replicates**

- You should report how often each experiment was performed
- You should include a definition of biological versus technical replication
- The data obtained should be provided and sufficient information should be provided to indicate the number of independent biological and/or technical replicates
- If you encountered any outliers, you should describe how these were handled
- Criteria for exclusion/inclusion of data should be clearly stated
- High-throughput sequence data should be uploaded before submission, with a private link for reviewers provided (these are available from both GEO and ArrayExpress)

Please outline where this information can be found within the submission (e.g., sections or figure legends), or explain why this information doesn’t apply to your submission:

The experiment was performed only once on each subject. There were no replicates for the purpose of this study. Hence, this column doesn’t apply to our submission.

There were a few subjects in whom longitudinal data was collected (annually) as part of a different study. However, we used the first year’s dataset for these subjects (unless that was not analyzable, in which case we chose the next best dataset for such subjects). We have provided the details in the Methods section.

**Statistical reporting**

- Statistical analysis methods should be described and justified
- Raw data should be presented in figures whenever informative to do so (typically when N per group is less than 10)
- For each experiment, you should identify the statistical tests used, exact values of N, definitions of center, methods of multiple test correction, and dispersion and precision measures (e.g., mean, median, SD, SEM, confidence intervals; and, for the major substantive results, a measure of effect size (e.g., Pearson's r, Cohen's d)
- Report exact p-values wherever possible alongside the summary statistics and 95% confidence intervals. These should be reported for all key questions and not only when the p-value is less than 0.05.

Please outline where this information can be found within the submission (e.g., sections or figure legends), or explain why this information doesn’t apply to your submission:

All our statistical interpretations were based on non-parametric tests on medians using Kruskal-Wallis Test (results were similar if we used Wilcoxon sign rank test). We used 2-way ANOVA for comparing change in alpha/gamma power across spatial frequencies and orientations for 227 healthy subjects (see Results section). We have reported exact (uncorrected) p-values for statistical tests. This information is provided at appropriate places in the Results section and in figure legends of figure supplements. We have further provided time-frequency plots for each MCI and AD subject individually in figure supplement 1 of Figures 2 and 3.

(For large datasets, or papers with a very large number of statistical tests, you may upload a single table file with tests, Ns, etc., with reference to sections in the manuscript.)

**Group allocation**

- Indicate how samples were allocated into experimental groups (in the case of clinical studies, please specify allocation to treatment method); if randomization was used, please also state if restricted randomization was applied
- Indicate if masking was used during group allocation, data collection and/or data analysis

Please outline where this information can be found within the submission (e.g., sections or figure legends), or explain why this information doesn’t apply to your submission:

This information can be found in the Methods section (participants sub-section). Detailed description of allocation into Healthy, MCI, AD groups could be found in Supplementary Tables 1-3 in Supplementary file 1.

**Additional data files (“source data”)**

- We encourage you to upload relevant additional data files, such as numerical data that are represented as a graph in a figure, or as a summary table
- Where provided, these should be in the most useful format, and they can be uploaded as “Source data” files linked to a main figure or table
- Include model definition files including the full list of parameters used
- Include code used for data analysis (e.g., R, MatLab)
- Avoid stating that data files are “available upon request”

Please indicate the figures or tables for which source data files have been provided:

All spectral analyses were performed using Chronux toolbox (version 2.10), available at http://chronux.org. Relevant data and codes are available at the following GitHub repository: <https://github.com/supratimray/TLSAEEGProjectPrograms>.
